# Developmental Changes in Children’s Processing of Redundant Modifiers in Definite Object Descriptions

**DOI:** 10.3389/fpsyg.2016.01900

**Published:** 2016-12-05

**Authors:** Ruud Koolen, Emiel Krahmer, Marc Swerts

**Affiliations:** Tilburg Center for Cognition and Communication, Tilburg UniversityTilburg, Netherlands

**Keywords:** language development, pragmatics, overspecification, maxim of quantity, referring expressions

## Abstract

This paper investigates developmental changes in children’s processing of redundant information in definite object descriptions. In two experiments, children of two age groups (6 or 7, and 9 or 10 years old) were presented with pictures of sweets. In the first experiment (pairwise comparison), two identical sweets were shown, and one of these was described with a redundant modifier. After the description, the children had to indicate the sweet they preferred most in a forced-choice task. In the second experiment (graded rating), only one sweet was shown, which was described with a redundant color modifier in half of the cases (e.g., “the blue sweet”) and in the other half of the cases simply as “the sweet.” This time, the children were asked to indicate on a 5-point rating scale to what extent they liked the sweets. In both experiments, the results showed that the younger children had a preference for the sweets described with redundant information, while redundant information did not have an effect on the preferences for the older children. These results imply that children are learning to distinguish between situations in which redundant information carries an implicature and situations in which this is not the case.

## Introduction

In referential communication, speakers often have communicative intentions that go beyond the identification of a target referent. For example, imagine a mother producing the following utterance to her young son: “Be careful with the big wine gum!”, in a setting where there is only one wine gum present. Obviously, by producing this utterance, the mother has the intention to communicate to her son that he has to be careful with the wine gum in order to prevent him from choking. In doing this, she uses two cues: firstly, she explicitly uses the imperative verb phrase ‘be careful,’ and secondly, there is an implicit cue, being the adjective ‘big.’ In the literal sense, this adjective is not necessary for unique identification of the wine gum, so that it can be considered to be redundant: there is no other wine gum at play, and the child will probably be able to estimate the size of the wine gum himself. Thus, at first sight, a description such as “the big wine gum” is *overspecified* in this example context.

Of course, in this particular situation, emphasizing the big size of the wine gum is driven by the mother’s communicative intentions: it will (hopefully) cause the child to reason that his mother includes this information to warn him against the hazards of choking on large objects. Following Grice (1975), this kind of implicit reasoning is triggered by a *conversational implicature*, resulting from a violation of the Gricean Maxim of Quantity. This maxim states that a contribution to a dialog should be as informative as required, but not more informative. In our example, the mother’s use of the adjective ‘big’ appears to violate the Maxim of Quantity, thereby triggering the implicit reasoning sketched above.

Redundant modifiers do not always evoke a specific implicature. Many previous studies have shown that speakers might also overspecify routinely, referring, say, to a single wine gum as “the red wine gum” because color is a salient attribute that attracts attention (Pechmann, 1989). The redundant modifier ‘red’ would probably not trigger a conversational implicature here, because listeners are generally able to distinguish redundant modifiers that yield extra connotations from redundant modifiers that do not serve such a pragmatic purpose. Actually, assigning special status to the redundant information in this example would be inconsistent with what the speaker intended to communicate.

While adult language users are generally well able to monitor each other’s implicit communicative intentions (e.g., Levinson, 2000), this might be different for children: younger children in particular are still learning to understand the implications of redundant information (Siegal and Surian, 2004), and to distinguish between situations in which speakers aim to elicit an implicature and situations where this is not the case. In this paper, we therefore study how children comprehend redundant information that does not serve any specific goal, such as is the case with ‘red’ in the example given above. Are children able to derive that the modifier is purely redundant? Or do they reason that the object’s color is relevant somehow? And how do children develop in this respect? We answer these questions by presenting two comprehension studies, where we used forced-choice tasks (Experiment 1) and graded rating scales (Experiment 2) to study the effect of redundant information on children’s preferences for sweets.

## Theoretical Background

Children start referring to physical objects in the world around them when they are around 12 months old, using first words (Fenson et al., 1994) and pointing gestures (Tomasello et al., 2007). Between the age of 2 and 4, more complex references are uttered, but these are often underspecified, and do not contain enough information to identify the target (Matthews et al., 2007, 2012). Later, around the age of 5, children become aware of the information they share with their listener (Nadig and Sedivy, 2002). Children start including redundant information regularly roughly from the age of 7 (Ford and Olson, 1975; Whitehurst, 1976), and they continue to do so as adults (e.g., Pechmann, 1989; Arts, 2004; Tarenskeen et al., 2015; Rubio-Fernández, 2016).

So why do adult speakers overspecify so often? As we have seen in the example with the mother and the wine gum, one reason is to evoke an implicature. However, one can also think of situations in which the redundant information does not serve a specific communicative purpose. In those cases, speakers seem to overspecify routinely, for speaker-internal reasons. Previous work in this direction has for example revealed that overspecification is triggered by the presence of visual clutter (Koolen et al., 2016), the incremental nature of speech production (Pechmann, 1989), and the amount of visual variation in the scene (e.g., Clarke et al., 2013; Koolen et al., 2013). In these cases, speakers do not necessarily take the listener perspective into account, but include redundant attributes that are perceptually salient and grab their attention.

The observation that overspecification is not always intended to evoke a specific implicature has at least one important implication: *listeners* must distinguish between situations where the redundant modifier communicates implicit extra information, and situations where this is not the case. Are they always able to do so in a successful way?

### The Cooperative Principle

For adult listeners, it can be argued that they have no difficulty in comprehending overspecified referring expressions. According to Grice’s (1975) Cooperative Principle, this is because experienced speakers and listeners tend to co-operate when they are in a conversation (Brennan and Clark, 1996). To characterize the expectations of people in a conversation, Grice introduced four maxims, which hold that speakers should not say less or more than is required (Maxim of Quantity), that they should tell the truth and avoid unfounded statements (Maxim of Quality), that their contribution should be relevant (Maxim of Relation), and that they should avoid obscurity and ambiguity (Maxim of Manner). These maxims have important implications for listeners (Grice, 1989), who might fail to understand the speakers’ communicative intention if one of the maxims is violated. In that case, the listener may draw a false implicature, or even no implicature at all.

Given that adult speakers are generally cooperative, it is plausible to assume that speakers aim to prevent their listeners from deriving false implicatures, and that they thus make sure that their listeners are able to assess the relevance of the information that they are provided with. Experimental findings from Engelhardt et al. (2006) are compatible with this assumption: although their study is not necessarily informative about speakers’ aims, it shows that adult listeners who are asked to judge the quality of instructions do not rate overspecified referring expressions lower than minimally specified ones. One explanation for this result could be that adult listeners are expected to have much linguistic experience (Hendriks, 2016), which makes them better able to judge whether a redundant modifier carries implicit meaning or not. However, for child listeners, the situation may be different: they might not understand why, when and how the conversational maxims are violated, and thus fail to understand a speaker’s communicative intentions (Siegal and Surian, 2004). In other words, children are expected to have a higher chance of deriving false implicatures – or no implicature at all – than adults.

In the next section, we discuss previous literature on children’s development in the derivation of two kinds of conversational implicatures related to Grice’s Maxim of Quantity. We report on existing research on how children of different age groups process redundant information during reference resolution, and during the comprehension of scalar implicatures.

### Children’s Ability to Master Quantity Implicatures

It is traditionally assumed that until the age of 7 or 8, children are not good at evaluating the communicative content of the expressions that they are presented with. Some early studies (e.g., Ackermann, 1981; Bonitatibus et al., 1988; Ackermann et al., 1990) argue that young children generally find it hard to distinguish between ambiguous and informative descriptions when selecting a target, in the sense that they have difficulty indicating whether their selected object is the “right one,” the one the speaker “meant,” or the one the speaker “could have meant.” Ackermann et al. (1990) explain this by claiming that young children often fail to derive the speaker’s communicative intentions from an expression, and relate it to the common ground that is shared between the speaker and the listener: children under the age of 8 find it difficult to infer relevant information from shared knowledge.

Some studies directly measure the impact of redundant attributes on child listeners. These studies investigate overspecification in the light of identification: to what extent do redundant attributes help or inhibit a child to select a target referent? For example, Sonnenschein (1982) found that redundant information facilitated target identification for 9-year-old children, but not for their 5-year-old counterparts. Sonnenschein explained these results by arguing that the memory capacity of younger children is not sufficient to process redundant modifiers. In a similar vein, Davies and Katsos (2010) applied a binary judgment task and magnitude estimation ratings to investigate whether 5-year-olds and adults perceived minimally specified object descriptions as more natural than overspecified ones. The results for the magnitude estimation task (but not for the binary task) revealed that the children indeed rated the overspecified descriptions lower than the minimally specified ones, which implies that children are already sensitive to violations of the Maxim of Quantity from the age of 5. Similar results were found for the adults, with lower ratings for overspecified descriptions rather than minimally specified ones. Note that this pattern for the adult participants is inconsistent with the pattern reported by Engelhardt et al. (2006), see Davies and Katsos (2013), and Engelhardt (2013) for a detailed discussion of this inconsistency.

Also Krahmer et al. (2013) used graded ratings to study the impact of redundant modifiers on children of different age groups. In this study, 6- and 9-year-old children were asked to estimate the size of a target referent (a toy) that was either referred to with a minimally specified description (e.g., the football) or an overspecified one, using a size attribute (e.g., the large football). The results again showed an effect of age group: the young children made larger size estimates than the older ones in the overspecification condition, but not in the minimal condition. According to Krahmer et al. (2013), these results suggest that 9-year-old children are less sensitive to redundant size modifiers than their 6-year-old counterparts.

Besides quantity implicatures in the context of reference resolution, children must also learn to derive scalar implicatures. Such implicatures follow from sentences in which scalar modifiers are used. For example, the sentence “Some toys are green” elicits the implicature that at least two toys are green, but not all. The traditional view on the derivation of scalar implicatures is that children are not able to comprehend scalar quantifiers at adult-like level until they are 7 years old (Noveck and Reboul, 2009). However, recently, this view has been nuanced: for example, children’s ability to master scalar implicatures depends on the type of quantifying term that they have to comprehend (Musolino, 2004; Geurts et al., 2010), and, more important for the current study, on the experimental task that they are faced with (Katsos and Bishop, 2011).

To investigate the impact of task, Katsos and Bishop (2011) presented 5-year-old children with videos in which a protagonist performed some course of action with a number of objects (e.g., a mouse picks up five carrots, but leaves five pumpkins unattended). After every video, the children had to judge (Experiment 1) or reward (Experiment 2) a statement. In half of the stimuli, the statements contained the scalar modifier ‘some,’ causing them to be pragmatically underinformative in the context of the performed action (e.g., “the mouse moves some of the carrots”). The other half of the stimuli contained statements that were fully informative. In the first experiment, Katsos and Bishop (2011) used a binary judgment task to let the children indicate whether the statements were right or wrong. They found that the participants rejected underinformative scalar expressions in only 26% of the cases, suggesting that children are not able to derive scalar implicatures related to ‘some’ at the age of 5. However, when the authors used three-point Likert scales to reward the statements (which they did in their second experiment), the underinformative statements were rated lower than fully informative ones. Katsos and Bishop (2011) explain these results by suggesting that the children’s poor performance in the first experiment was due to the binary task, and that children are already sensitive to violations of the Gricean maxims at the age of 5.

## The Current Study

In the previous sections, we have described how children of different ages develop their ability to successfully derive implicatures related to the Maxim of Quantity (Grice, 1975). The picture that emerges from the literature raises two interesting observations. Firstly, some previous work has shown that children are not sensitive to violations of the Maxim of Quantity at the age of 5 (e.g., Sonnenschein, 1982; Noveck and Reboul, 2009; Krahmer et al., 2013), while other work has shown that they are (e.g., Davies and Katsos, 2010; Katsos and Bishop, 2011). Secondly, the experimental task that is used affects the children’s performance to a large extent, in such a way that it has been shown that 5-year-olds do not reject redundant (Davies and Katsos, 2010) or underspecified (Katsos and Bishop, 2011) descriptions in a binary judgment task, but that they do so when graded rating scales are used.

The current study aims to gain further insight in these two issues by addressing the question how children develop their ability to process overspecified descriptions in situations where the speaker includes a redundant attribute in a description, but *not* with the purpose to elicit a specific implicature. This is an important question, since speakers may overspecify for various reasons. On the one hand, speakers may overspecify “on purpose” to elicit a more traditional Gricean implicature. This is what happens in the example where a mother mentions the size of a sweet to warn her child. On the other hand, as noted earlier, redundant attributes can also be used routinely: speakers may, for example, use redundant attributes that are visually salient and grab their attention, such as color (e.g., Pechmann, 1989; Koolen et al., 2013). For children, the question is whether they are able to monitor the speaker’s communicative intentions: at some point in development, they must learn to decide when a redundant attribute is relevant in a given communicative context, and when it is not.

In our experiments, we present children with overspecified descriptions of sweets, and test to what extent the redundant information determines their preferences for the sweets. We compare the performance of children of two age groups (6- and 7-year-olds, and 9- and 10-year-olds), and hypothesize that the younger children are affected by the redundant information in their preferences for sweets, but that the older children are not. Our reasoning is that older children have learned to distinguish between situations in which speakers aim to elicit an implicature and situations in which this is not the case, while younger children might overgeneralize by reasoning that redundant information is *always* used to trigger an implicature. In the case of overgeneralization (which is a well-known phenomenon in child language acquisition; Tomasello, 2003), a child will wonder about the speaker’s intention with including the redundant attribute. However, given that there is none, the child may reason that the speaker intends to draw special attention to the sweet, for example to communicate that it should be preferred and that it is the right choice in the current situation. One alternative hypothesis would be that any effect of redundant information is due solely to attentional asymmetries, and to children’s capacities to switch attention between different objects. In the general discussion, we elaborate further on the role of attention.

Because experimental task might affect children’s performance (Davies and Katsos, 2010; Katsos and Bishop, 2011), we present the results of two experiments in which we test the above hypothesis: one using a binary forced-choice task and one using 5-point rating scales. We manipulate color and shape modifiers as redundant information.

## Experiment 1: A Forced-Choice Task

In our first experiment, we presented children of two age groups with pictures of two sweets. In all critical trials, these two sweets were identical, and one of them was referred to with a redundant color or shape modifier. In a forced-choice task, the children were asked to indicate which of the two sweets they preferred, based on the descriptions they had heard.

### Method

#### Participants

Participants were 49 children of two age groups. The sample of younger children consisted of 22 children (10 males, 12 females) with a mean age of 6;7 years (ranging between 6;0 and 7;3). The sample of older children consisted of 27 children (12 males, 15 females) with a mean age of 9;8 years (ranging between 9;2 and 10;2). All children were recruited at the same primary school, and had Dutch as their primary language. They had been given permission to participate by their parents via a signed consent form.

#### Materials

**FIGURE 1:**
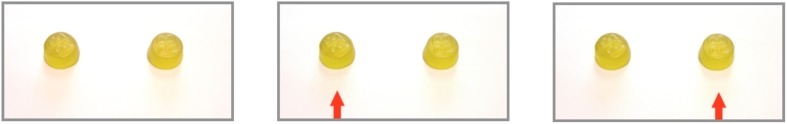
**An example of a critical trial.** The trial started and ended with the left picture. In between, the middle and right pictures were presented (highlighting the left and right sweet).

The materials consisted of pictures of two sweets. These two sweets were placed next to each other. In the critical trials, the two sweets were of the same kind and color (see the left picture in **Figure 1**), which meant that the participating children had no reason to have an *a priori* preference for one of the two.

Pre-recorded descriptions of the two sweets were played while the pictures were shown to the children. In every trial, one of the sweets (either the left or the right one) was referred to with a redundant modifier; the other sweet was simply referred to as “this sweet.” The spoken descriptions were presented as questions that had the following basic structures, depending on which sweet was referred to with a modifier: “*Would you like this (…) sweet or this sweet?*” or “*Would you like this sweet or this (…) sweet?*” First, a picture of the two sweets was shown (see the left picture of **Figure 1**), during which the first part of the question was played: “*Would you like…*”. After that, the description of the first (left) sweet was played. During this description, the corresponding sweet was highlighted with a red arrow (see the middle picture of **Figure 1**). Once the left sweet had been described, a description of the right sweet followed, again highlighted with a red arrow (see the right picture of **Figure 1**). After the two descriptions, the child had to indicate which sweet he or she preferred. The pre-recorded descriptions were produced by a female voice with a natural intonation, to avoid overly contrastive accents that would put (too) much emphasis on the redundant modifier. Note that the combination of the arrow with the basic description “*this sweet*” was always sufficient to unambiguously identify the sweet, i.e., the inclusion of a modifier always resulted in an overspecified description.

There were six critical trials in which the redundant modifier provided perceptual information about one of the two sweets. In four critical trials, information about the color of the sweets was used, while shape information was provided in another two critical trials. For example, in the critical trial depicted in **Figure 1**, this led to the following question: “*Would you like this sweet or this yellow sweet?*” Based on the descriptions, the children were asked to indicate which sweet they preferred. Whether the left or right sweet was described with a redundant modifier was counterbalanced over trials.

We performed a pre-test in order to make sure that the children were aware that the two sweets in the pictures used for the critical trials looked in fact identical. To do this, we presented six pictures of two identical looking sweets (the same as the ones used in our experiment) and six pictures of two different sweets (randomly selected from the fillers) to 20 children (ten from each age group). None of these children had participated in the main experiment. For each picture, we asked the children the following question: “*Do these two sweets taste the same?*” For the pictures of identical sweets, the results showed that the children expected the two sweets to taste identical in 100% of the cases, and this percentage decreased to 2% for the pictures of different sweets. We conclude from this pre-test that all information that was provided in speech about the sweets in the critical trials could be considered redundant. Hence, if a child preferred the redundantly described sweet, the redundant modifier arguably guided the choice for this sweet.

Sixty-six trials were included as fillers, the majority of which (48) consisted of pictures of two different kinds of sweets, both described with various kinds of information (a combination of perceptual and affective information). A small minority of the fillers (18) consisted of pictures of two similar sweets. In the majority of these trials, one of the two sweets was described with affective information (such as ‘delicious’ or ‘disgusting’). The remaining six fillers served as a baseline, allowing us to check whether the children were biased in favor of either the left or the right sweet. In these cases, neither of the two sweets was described with a modifier, and the following question was asked: “*Would you like this sweet or this sweet?*” The results showed that the children did not have a bias for left or right: the younger children chose the left sweet in 50% of the baseline trials, while the older ones did this in 51.8% of the cases. These proportions did not differ significantly from chance level (i.e., 50%), as revealed by one-sample *t*-tests that were applied to the participants’ mean scores on the six baseline trials [young children: *t*(21) = 0.00, *ns*; older children: *t*(26) = 0.035, *ns*]. Based on these results, we ignore order of presentation in the analyses of the data.

#### Procedure

The procedure was identical for the children in both age groups. The experiment had a running time of approximately 15 min, and was individually performed in a quiet room inside a school building. The experiment was conducted in Dutch. We created one block of 72 trials in a fixed random order (this was presented to one half of the participants), and a second block containing the same trials in reverse order (which was presented to the other half of the participants). Our analyses did not show a significant difference between these two groups, meaning that there was no evidence of an effect of block on children’s choices for sweets.

After a child had entered the experimental room, he or she was asked to take place in front of a computer screen. The experimenter was seated next to the child for the duration of the experiment. The instructions (which were provided orally by the experimenter) explained that the children first had to listen carefully to the pre-recorded descriptions that were given about the two sweets, and that they then had to indicate which of the two sweets they preferred. They could either do this by pointing at the sweet of choice on the screen, or by telling the experimenter which choice they had made. It was emphasized that the children had to base their choices on the descriptions that were provided about the sweets. Each time a child had completed a trial, the experimenter marked his or her choice on an answering form. In each trial, the children had 4 seconds to indicate which sweet they preferred, but they were given more time in case this was necessary. The experimental procedure started with three practice trials to acquaint the children with the procedure. After the completion of the experiment, the children confirmed that they had understood the experimental task. When explicitly asked, none of the children indicated to have been aware of the actual goal of the study.

#### Design and Statistical Analysis

The experiment had a between-participants design with *age group* (levels: young, old) as the independent variable, and the proportion of choices for the redundantly described sweet as the dependent variable. The younger children in the sample were 6 or 7 years old, and the older children were 9 or 10 years old. In order to test for significant differences between the two age groups, we performed a one-way ANOVA. In order to control for departures of normality, we also ran the ANOVA on arcsin transformed proportions. Because the results for this additional analysis showed exactly the same picture as those for the untransformed data, we stick to the untransformed data in our results section. We applied one-sample *t*-tests on the participants’ proportions to see whether these were significantly different from chance level (i.e., 50%) in either of the two age groups.

### Results and Interim Discussion

**FIGURE 2:**
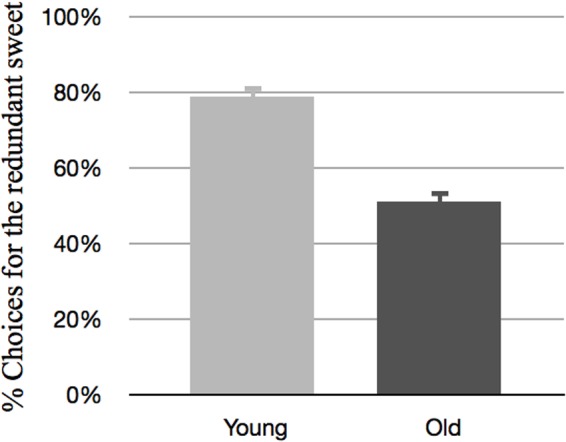
**The proportion of choices for the redundantly described sweets as a function of age group**.

**Figure 2** depicts the proportion of choices for the sweets that were described with redundant information as a function of the two different age groups.

**Figure 2** shows a clear difference between the younger and older children in terms of the proportion of choices for the sweets that were described with a redundant modifier. As reflected in an effect of *age group* [*F*_(1,47)_ = 35.22, *p* < 0.001, η^2^ = 0.43], the young children were far more sensitive to these redundant modifiers. More specifically, we found that the young children were more likely to choose the sweet that was described with redundant information (*M* = 0.79, *SD* = 0.03), and this proportion was significantly higher than 50% chance level: *t*(21) = 9.80, *p* < 0.001. For the older children, however, the results showed a different pattern, in the sense that they only chose the redundantly described sweet in around half of the cases (*M* = 0.51, *SD* = 0.03). This proportion was not significantly different from 50% chance level: *t*(26) = 0.36, *ns*.

The above results indicate that while younger children are guided in their choices by redundant modifiers, older children are not. For the older children, we found that they were not sensitive to redundant modifiers related to the color or shape of the sweets: in the cases where they had to choose between for example “the green sweet” and “the sweet”, they scored at chance level. This was not the case for the younger children, who chose the redundantly described sweet in almost 80% of the cases.

As we have seen in the introduction section, earlier work has emphasized the role of the experimental task if one wants to study children’s ability to successfully derive quantity implicatures. Particularly, in some situations, 5-year-olds have been shown to perform better when magnitude estimation scales rather than binary judgment tasks are used (e.g., Davies and Katsos, 2010; Katsos and Bishop, 2011). Therefore, in the next experiment, we replicate our first experiment with pictures of single sweets. Instead of binary judgments, we use 5-point rating scales to measure to what extent children are affected by redundant information in their ratings. The use of graded rating scales has the additional advantage that the task becomes somewhat more natural, since the children no longer have to choose between two identical looking sweets (see below).

## Experiment 2: Graded Rating Scales

### Method

#### Participants

Participants were 60 children in roughly the same age groups as those used in the first experiment. The sample of younger children consisted of 30 children (15 males, 15 females) with a mean age of 7;1 year (ranging between 6;6 and 8;3). The sample of older children consisted of 30 children as well (17 males, 13 females) with a mean age of 10;2 years (ranging between 8;7 and 11;5). The children had not participated in the previous experiment, had Dutch as their primary language, and had been given permission to participate by their parents via signed consent.

#### Materials

**FIGURE 3:**
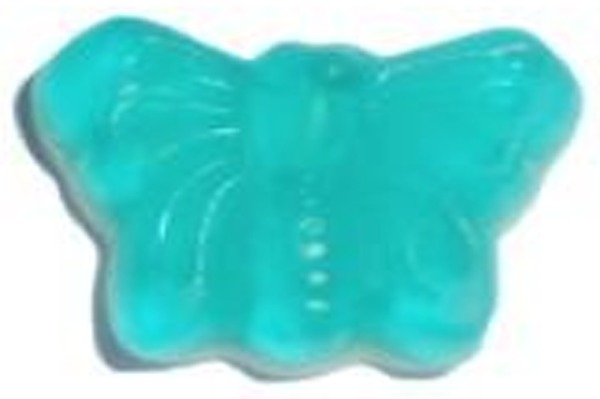
**Example of a critical trial in the second experiment.** A pre-recorded description of the sweet was played while a trial was shown.

Experiment 2 was a partial replication of Experiment 1, in the sense that we again measured children’s preferences for sweets, but this time only one sweet was depicted in every trial (see **Figure 3**). In all trials, a pre-recorded description of this sweet was played while it was shown. The descriptions were produced by a male voice using a natural intonation, again avoiding (too) prominent accents. Since there was only one sweet in every picture, the basic description “*The sweet*” was always sufficient for unambiguous identification, causing any modifier to be redundant.

This second experiment had 12 critical trials in two conditions. In the unmodified condition (represented by six trials), the depicted sweet was simply described as “*The sweet*”. The other six critical trials formed the overspecification condition. As in Experiment 1, the sweets in this condition were described using perceptual information, but now only color modifiers were used. For example, in **Figure 3**, this would lead to a description such as “*The blue sweet*.” In order to avoid that *a priori* preferences for sweets would interfere with our findings, the same six pictures of sweets were used in both conditions.

**FIGURE 4:**
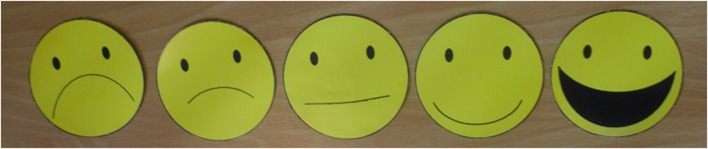
**The cardboard smiley faces representing the 5-point rating scale**.

The crucial difference between Experiments 1 and 2 was related to the way in which the children marked their preferences for sweets in every trial: while a forced-choice task was used in the first experiment, we now used 5-point graded rating scales. This went as follows: after having been presented with a picture of a sweet and having listened to the corresponding description, the children were asked to indicate to what extent they liked the sweet. This was done via a pre-recorded question (“*How much do you like this sweet?*”), which was played automatically after the description of a sweet, and was the same for all trials. The children could indicate the extent to which they liked the sweet by pointing at one of the cardboard smiley faces that were lying in front of them. Such facial representations of graded rating scales are commonly used in studies with children (e.g., Lockl and Schneider, 2002; Visser et al., 2014). As **Figure 4** shows, the smiley faces represented five categories, ranging from “not at all” to “very much.”

As fillers, we used pictures of sweets and other kinds of food (such as bananas and eggs). As in the critical trials, always one item was depicted per trial. These items could be described by means of an affective modifier (such as delicious or disgusting), or via a combination of affective and perceptual information. Thirty-six fillers were included, resulting in a total of 48 trials for the entire experiment.

#### Procedure

The procedure of the current experiment was essentially similar to the one followed in the first experiment, and was the same for the children in the two age groups. The experiment was conducted in a quiet room in a school building, and had an average running time of approximately 10 min. The language of the study was Dutch. The trials were presented in a fixed random order, which was the same for all children in both age groups.

The children were seated in front of a computer screen depicting the trials, with the experimenter seated next to the child for the course of the entire experiment. As in the first experiment, the instructions were provided orally, and it was explained that the children had to listen carefully to the sweet descriptions. Moreover, the 5-point graded rating scale was introduced, and the children were explicitly asked to base their choices on the pre-recorded descriptions of the sweets. Two practice trials were included to acquaint the children with the rating scale.

After every trial, the experimenter marked which of the five smiley faces the child had pointed at on an answering form. Like in the previous experiment, the children had 4 seconds to make their choice, but more time was given if necessary. Afterward, the children confirmed that they had understood the experimental task. Most of them indicated that they thought that the task was about food in general.

#### Design and Statistical Analysis

The experiment had one between-participants variable (*age* – levels: young and old), and one within-participants variable (*condition* – levels: unmodified and overspecified). The dependent variable was the extent to which the children liked the sweets, with scores ranging from 1 to 5. The younger children in the sample were again 6 or 7 years old, and the older ones were again 9 or 10 years old. We performed a mixed ANOVA to test for significance. The analysis was done at the level of the individual trials.

### Results and Interim Discussion

**FIGURE 5:**
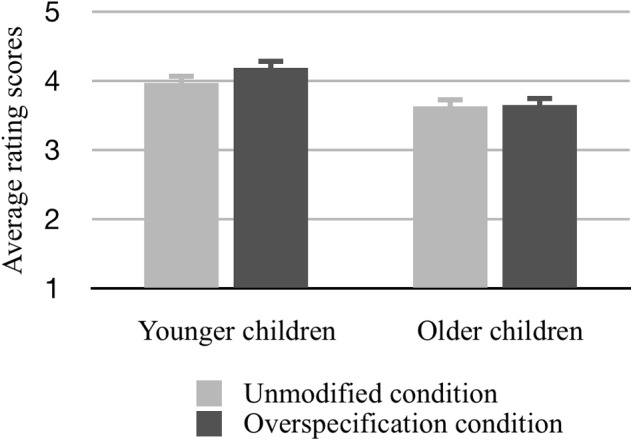
**The average scores of the children in the two age groups, as a function of the unmodified and the overspecification conditions**.

**Figure 5** depicts the average scores of the younger and older children as a function of the two conditions.

Firstly, the results revealed a main effect of *age* [*F*_(1,58)_ = 6.07, *p* < 0.02, η^2^ = 0.10], showing that the younger children (*M* = 4.08, *SD* = 0.13) generally liked the sweets better than the older children (*M* = 3.64, *SD* = 0.13). Furthermore, there was a main effect of *condition* [*F*_(1,58)_ = 3.98, *p* = 0.05, η^2^ = 0.06], indicating that the scores in the trials where a sweet was described with redundant information (*M* = 3.91, *SD* = 0.09) were significantly higher than the scores in the unmodified condition (*M* = 3.81, *SD* = 0.09). As **Figure 5** suggests, we also found a significant interaction between *age* and *condition* [*F*_(1,58)_ = 6.00, *p* < 0.02, η^2^ = 0.09], showing that the effect of condition was due to the performance of the younger children: while the older children gave practically equal scores to the sweets in the unmodified (*M* = 3.63, *SD* = 0.13) and the overspecification (*M* = 3.65, *SD* = 0.13) conditions, the younger ones liked a sweet better when it was described with a perceptual modifier (*M* = 4.19, *SD* = 0.13) than when no modifier was used (*M* = 3.97, *SD* = 0.13).

**FIGURE 6:**
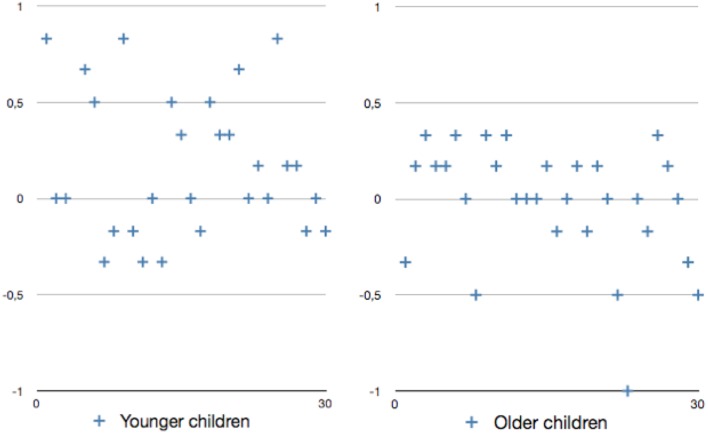
**Speaker variation in the two age groups: the difference in the scores for the unmodified and the overspecification conditions (*y*-axis) as a function of the individual children in the two age groups (*x*-axis).** A positive value indicates that a child rated the overspecified descriptions higher than the minimal ones.

The above interaction between age group and condition follows a similar pattern as compared to the results of the first experiment (in which we also found differences between the unmodified condition and the overspecification condition for the younger children, but not for the older ones). However, this time, the effect sizes that we measured were generally smaller. Therefore, we focused on the individual scores for all participants, to see whether the effects of redundancy were consistent across participants in either of the age groups. In doing so, for each child we calculated the difference between the scores in the overspecification condition and the unmodified condition. For example, if a child scored 4.15 on average in the overspecification condition and 3.95 on average in the unmodified condition, the individual score for this child was 0.20. **Figure 6** depicts the individual scores for all children that took part in this second experiment.

As can be seen in **Figure 6**, the number of children who scored zero (7 younger children; 8 older children) or below zero (8 younger children; 9 older children) was practically equal for the two age groups, which shows that the differences between conditions that we observed are not consistent across all children in the two age groups. Hence, only eight children in the younger group scored 0.5 or higher, which is in line with the fact that the effect sizes in this second experiment were rather small. Finally, we observe that the amount of individual variation between children was greater in the younger group than in the older group. We come back to this issue of individual variation in the Section “General Discussion.”

The results of the second experiment again indicate that 6- and 7-year-old children are guided in their preferences by redundant information to a larger extent than 9 and 10-year-old children. In particular, the younger children liked a sweet significantly better if it was described with a (redundant) color attribute than in the unmodified case, while the scores of the older children were practically identical in both conditions. Although a different task was used, these results show the same pattern as those found in the first experiment. However, note that the effect sizes that were measured here were generally smaller.

## General Discussion

The results of the two experiments presented in this paper revealed that 6- and 7-year-old children are influenced by redundant color and shape modifiers in their preferences for sweets, while 9- and 10-year-old children in a similar setting generally ignore such information. The observed age differences are consistent across two experimental tasks: a binary forced-choice task (Experiment 1), and a task that used 5-point graded rating scales (Experiment 2). Our results are in line with our hypothesis, and suggest that younger children process redundant information in a different way than older children. How can we explain this difference?

To answer this question, we would like to take the reasoning behind our hypothesis as a starting point. Naturally, we distinguished between different reasons why speakers may overspecify: sometimes, they include a redundant modifier on purpose in order to elicit a specific implicature, but they may also use redundant information routinely, for example because certain target attributes attract their attention (e.g., Pechmann, 1989; Krahmer et al., 2013). We expected to find that the older children in our sample would be able to distinguish between these two scenarios, while the younger children would not be able to do so, or at least not to the same extent. This expectation was grounded in previous literature on language acquisition, which shows that children do not start to overspecify their own object descriptions regularly until they are 7 years old (Ford and Olson, 1975; Whitehurst, 1976), and that the age of 7 also marks the point from which they should be able to comprehend scalar quantifiers at adult-like level (Noveck and Reboul, 2009). In addition, previous research showed that 5-to-6-year-olds have a hard time comprehending overspecified descriptions, while 9-year-olds and adults do not have these problems (Sonnenschein, 1982; Krahmer et al., 2013). According to Siegal and Surian (2004), such differences between age groups can be attributed to pragmatic development: children must simply learn to interpret the pragmatic implications of the redundant information that is provided to them in a conversation.

Following Siegal and Surian’s (2004) argumentation, pragmatic development might also explain the age differences observed in the current paper: after all, the children in our experiments had to judge whether the redundant modifiers they were presented with were relevant in the context. Hence, if pragmatic development indeed explains our findings, then what is the nature of this development in Gricean terms? Based on the differences between the age groups, one may argue that older children have learned to distinguish between the various reasons why speakers may overspecify their object descriptions, and that redundant information does not necessarily carry an implicature. For example, in Experiment 1, our pre-test revealed that both the younger and older children considered the two sweets they were presented with to be similar. However, only the older children showed awareness that the color and shape information was not intended to evoke a specific implicature: their preference to choose redundantly described sweets was not different from 50% chance level.

For the young children, the situation was different: they showed a strong preference for sweets described with a redundant modifier (in Experiment 1), and also rated these sweets higher than the sweets that came with a minimal description (in Experiment 2). These results suggest that young children must still learn that violations of the Maxim of Quantity are only sometimes intended to elicit an implicature. Instead, children may overgeneralize, reasoning that redundant modifiers are *always* used to evoke a specific implicature. Such overgeneralization processes are ubiquitous in language acquisition. For example, the pre-emption hypothesis (Goldberg, 1995) states that there is a one–one correspondence between linguistic forms and meanings: once a child has learned a form for expressing a given meaning, other linguistic forms with the same meaning are discarded, unless other language input offers positive evidence for a second form. This prediction is in line with the entrenchment hypothesis (Braine and Brooks, 1995), which states that the meaning with the greatest overall frequency for a certain linguistic form blocks the assignment of a ‘new’ meaning. These kinds of usage-based processes have for example been found to occur when children learn to use certain adjectives (Boyd and Goldberg, 2011), and predict that relatively frequently used expressions might become highly predictable (Tomasello, 2003).

Applied to our findings, one model of pragmatic development based on usage-based processes may be that in order to learn that not all modification is intended to evoke a specific implicature, children need to be exposed to a critical mass of redundant modifiers, uttered with diverse communicative intentions. This way, due to increasing linguistic experience, they learn when to draw an implicature, but also to recognize the situations in which they should not do so. This line of reasoning may in particular be true for absolute modifiers such as color, since these are very frequently used as a result of speaker-internal, saliency-based processes (e.g., Pechmann, 1989; Koolen et al., 2013), without the intention to evoke a specific implicature.

What meaning did the younger children assign to the redundant modifiers that they were presented with, if they indeed overgeneralized and assumed that the overspecified descriptions were intended to elicit an implicature? We believe that the children first wondered (either implicitly or explicitly) about the implicit intentions of the speaker to use a redundant modifier. However, given that there were no clear underlying intentions, the children might have reasoned that the speaker simply intended to draw special attention to one sweet, to communicate that the sweet should be preferred and that it was the right choice in the communicative context. The reason why a redundant modifier made a sweet more attractive, and not less attractive, might be that children’s feelings about sweets in general are almost always positive by nature. Thus, if they indeed reasoned that the sweet with the modified description was the right choice in the communicative context, this might have caused their feelings about sweets to become even more positive.

If we assume that the younger children in our experiments indeed derived an implicature when a redundant modifier was used, it becomes particularly interesting to take a closer look at the individual differences that we observed Experiment 2. As depicted in **Figure 6**, the amount of variation between the scores for the younger children was substantial: for a minority of children, the difference between the scores in the overspecification condition and the unmodified condition was high, while for others it was non-existent. One interpretation of this pattern would be that only the children with the higher differential scores overgeneralized and derived an implicature, while the children who scored zero (or even negatively) were ahead in development and showed performance that was similar to the children in the older age group. Another possibility would be to explain the individual variation by means of a difference in cognitive capacity, akin to a recent cognitive model proposed by Hendriks (2016). In her model, Hendriks (2016) predicts that at least some of the variation between language users during reference production and comprehension should be explained by differences in working memory capacity and processing speed. Since working memory capacity and processing speed increase through maturation and linguistic experience (Hendriks, 2016), the children with low differential scores in **Figure 6** may have had insufficient cognitive resources available to derive an implicature, unlike the children with a higher score. To find empirical support for one of these explanations, it would be relevant to replicate the current study with even younger children, to see how they perform in the tasks at hand.

An alternative and perhaps simpler explanation for the observed age differences could be that the children did not even wonder about a specific implicature that the speaker intended to elicit, and that their preferences for sweets were determined solely by attentional asymmetries. This explanation suggests that the redundant modifier simply caused one sweet to be more prominent than the other, but only for the younger children. The effect of age group may in this scenario be due to a difference in children’s attentional capacities. After all, at least in Experiment 1, the task required some attention switching between the two sweets that were depicted in order to compare them. Given that one of the sweets was described with a longer NP, it could be that the younger children found it harder to switch their attention back to the other sweet once it was attracted to the sweet with the longer description. Related findings from the attention literature confirm that the process of attention switching gets easier and more flexible as children grow older (e.g., Hanania and Smith, 2010). However, it should be noted that attention switching was not required in our second experiment, where every trial depicted only one sweet. In future research, it would be thus interesting to study the role of attention in more detail, for example by replicating the first experiment in an eye-tracking paradigm. One could then analyze the number of attention switches as a function of age group. Hence, also measuring reaction times could serve to distinguish between pragmatic and attention-based explanations. Arguably, it may take some time to derive an implicature, so we believe that longer reaction times for pictures where one sweet is described with a redundant modifier would speak against the attention-based explanation.

Another issue that we raised in the Introduction section was related to the experimental task. Previous work found that the task affects young children in the ability to derive quantity implicatures. In particular, it was found that 5-year-old children do not reject redundant (Davies and Katsos, 2010) or underspecified (Katsos and Bishop, 2011) descriptions in a binary judgment task, but that they tend to do so when graded rating scales are used. In order to explain these differences, Katsos and Bishop (2011) introduced the Pragmatic Tolerance Hypothesis, which holds that certain tasks mask children’s actual competence of the Gricean Maxim of Quantity. Katsos and Bishop (2011) found evidence for this hypothesis by studying children’s ability to master scalar quantifiers such as “some.” However, it is interesting to speculate about the predictions of Pragmatic Tolerance for the quantity implicatures studied here.

The most straightforward prediction of the Pragmatic Tolerance Hypothesis for our experiments would be that redundant modifiers guide 6- and 7-year-old children in their preferences for sweets in Experiment 1 (where we used a forced-choice task), but that this effect should disappear in Experiment 2 (where graded ratings were used). At first sight, our findings speak against this prediction: the pattern that we observe in the second experiment is similar to that in the first experiment. However, a closer look at the effect sizes indicates that Pragmatic Tolerance might still play a role, as the effects of redundancy were less pronounced in the second rather than the first experiment. This difference seems to be in favor of the Pragmatic Tolerance Hypothesis: although the effects of redundancy did not disappear in Experiment 2, the experimental task has caused the children to be somewhat less tolerant toward overspecification there.

One final issue that we would like to discuss is whether different kinds of redundant modifiers have different effects on listeners. As described earlier, we manipulated both color and shape information in the overspecification condition in Experiment 1. Based on previous research with adult listeners, one may expect to find an effect of modifier type there. For example, an eye-tracking experiment conducted by Sedivy et al. (1999) revealed that participants were quicker to comprehend expressions such as *Pick up the tall glass* when another contrasting object of the same category (e.g., a small glass) was present in the context. However, for color attributes, a similar effect could not be found (Sedivy, 2003), which suggests that redundant color terms do not necessarily trigger contrastive inference. When we look at the percentage scores for the older children in our first experiment, we observe a pattern that seems to confirm this suggestion: shape modifiers (56%), but not color modifiers (49%), caused these children to have a slight preference for the redundantly described sweets over the sweets that came with an unmodified description. However, for the younger children, we see the opposite pattern: at this age, color (83%) had a bigger impact than shape (71%). Although we only used shape in two trials, and color in four, these percentages again hint at some form of development, as performance seems to become more adult-like as children grow older.

## Conclusion

In two experiments, we found that 6- and 7-year-old children are guided by perceptual redundant modifiers when indicting their preferences for sweets, while this was not the case for 9- and 10-year-old children. In particular, the younger children liked a sweet that was redundantly referred to as, say, “green,” better than the older children, irrespective of the task that was used to study these preferences. We reason that between 6 and 10 years of age, children learn that redundant modifiers are only sometimes intended to elicit a specific conversational implicature.

## Ethics Statement

All children that participated had been given permission to participate by the director of their primary school, and by their parents via a signed consent form. Before data collection, children were given an oral description of the task, plus an explanation that they were free to stop the testing at any given point. The measures that were going to be used were explained.

## Author Contributions

This manuscript is the result of a close collaboration between the three authors. The corresponding author RK has taken the lead in general, but also the second EK and third MS authors have contributed substantially to all stages of the research.

## Conflict of Interest Statement

The authors declare that the research was conducted in the absence of any commercial or financial relationships that could be construed as a potential conflict of interest.
